# Parental perspectives on support for learners with physical disabilities at special schools

**DOI:** 10.4102/ajod.v14i0.1640

**Published:** 2025-08-13

**Authors:** Makwena M. Sibuyi, Desmond Mathye, Muziwakhe D. Tshabalala, Komane Matthews Mphahlele, Nombeko Mshunqane

**Affiliations:** 1 Department of Physiotherapy, Faculty of Health Science, Sefako Makgatho Health Science, Tshwane, South Africa; 2 Department of Physiotherapy, Faculty of Health Science, University of Pretoria, Tshwane, South Africa; 3 Department of Inclusive Education, Faculty of Education, University of Limpopo, Polokwane, South Africa; 4 Department of Physiotherapy, Faculty of Health Science, University of KwaZulu-Natal, Durban, South Africa

**Keywords:** special schools, parents’ perspectives, learners with physical disabilities, curriculum differentiation, vocational education

## Abstract

**Background:**

Inclusive education for learners with special education needs is challenged with a rigid curricula and inadequate policy monitoring. Parental perspectives are crucial for shaping inclusive policies. However, these are insufficiently examined in the existing research and hinder improvements in special education practices.

**Objectives:**

This study explored parents’ perspectives on how special schools met the unique needs of learners living with physical disabilities.

**Method:**

A descriptive, qualitative exploratory design utilising semi-structured interviews with 11 parents from three selected special schools was adopted. Participants were recruited using a purposive non-random sampling method through telephone calls and face-to-face interviews. Transcripts were audio recorded and transcribed verbatim. Data were analysed inductively using a six-step approach to thematic data analysis on ATLAS.ti version 9. Intercoder reliability was achieved with consensus agreement.

**Results:**

Three themes emerged: (1) A lack of curriculum differentiation and its effects on the learners’ academic performance. (2) A lack of empathy and support in addressing the learners’ challenges. (3) Poor management of assistive devices.

**Conclusion:**

Parents perceived that special schools inadequately addressed their children’s needs, particularly in curriculum delivery. Insufficient assessments resulted in learners remaining in unsuitable academic stream instead of transitioning to vocational pathways.

**Contribution:**

Parental insights highlight critical areas for improvement in informing policies to enhance support for learners with special education needs.

## Introduction

Sustainable Development Goal 4 pronounces a global commitment to ensuring inclusive, equitable and quality education for all, with an emphasis on promoting lifelong learning opportunities (United Nations 2015). At its core lies a moral imperative that every child has the fundamental right to an education of high-quality that is responsive to their individual needs. The United Nations Educational, Scientific and Cultural Organization (UNESCO) Global Education Monitoring Report (2020) advocates for empathy-driven leadership and education systems that value diversity as key drivers of inclusion. Cultivating inclusive classrooms that recognise, and respect diverse learner experiences is foundational to building environments where all learners feel supported.

However, persistent challenges prevent the realisation of equitable education. These challenges include (but not limited to) inadequate support and training for educators and infrastructural inadequacies that fail to accommodate diverse learning needs (UNESCO 2020).

### The Three Stream Model of education

The South African Department of Basic Education (DBE) adopted the Three Stream Model of education as a strategy to provide learners with greater choice and flexibility, catering to their interests and abilities, while responding to learner attrition, low matriculation pass rates, and rising youth unemployment (DBE 2023).

The three streams refer to the academic, technical-vocational and occupational stream found within the National Curriculum Statement for Grades R-12. The academic stream is the traditional route focusing on theoretical knowledge, which prepares students for higher education. The technical-vocational stream emphasises practical skills and provides opportunities for students to acquire technical knowledge. The occupational stream targets skills-based learning and is designed to prepare students for direct entry into the workforce or entrepreneurship, especially for those who might struggle in the traditional academic route (Equal Education 2018). For these learners a Differentiated Curriculum and Assessment Policy Statement (DCAPS) curriculum is provided for in the foundation and intermediate phases (DBE 2021).

### Differentiated curriculum and teaching strategies

The DCAPS curriculum stands out from South Africa’s standard Curriculum and Assessment Policy Statement (CAPS) curriculum by placing stronger emphasis on practical skills. In Indonesia, the curriculum gives slightly more weight (60%) to vocational training than academic subjects, prioritising hands-on learning for students (Wijaya & Huda 2018). By comparison, South Africa places even greater focus (80%) on developing learners’ practical abilities over theoretical instruction (Shaffeei, Razalli & Hanif 2020; Zhang 2009). Despite differences in balance, both systems aim to support students who struggle with traditional academics through subjects such as woodwork and welding.

Vocational options are selected based on the school’s resources and the specific needs of learners to help them gain relevant workplace experience (DBE 2018; Ratnengsih 2017). To support this, educators must understand each learner’s strengths and challenges and use varied teaching methods to encourage active participation in the classroom (Reis & Renzulli 2018). Building rapport with students and collaborating with support staff are also essential to successful implementation (Tjernberg & Mattson 2014).

In Indonesia, having trained vocational educators and integrating these subjects into the timetable has helped students gain work experience through partnerships with Non-governmental organisations (NGOs) and businesses (Pasaribu & Harfiani 2021). In contrast, South African schools often face obstacles such as unskilled teachers, lack of curriculum specialists and inadequate infrastructure, making it difficult to offer a differentiated and responsive curriculum (Du Plessis 2020; Maniram 2015). These challenges highlight the need for well-designed support systems and professional development (Solomon, Luger & Ned 2024). Ultimately, the success of the DCAPS curriculum depends on educators’ ability to adapt academic content and deliver practical skills training that meets diverse learner needs (Akrim & Harfiani 2019; Reis & Renzulli 2018).

### Support envisaged at special schools for learners with special needs

Special schools play a key role in helping learners with special needs, especially when mainstream schools cannot offer the support they require. The National Policy on Screening, Identification, Assessment and Support (SIAS) and the National Policy on the Programme of Progression and Promotion Requirements (NPPPR 2017) guide schools on how to identify learning challenges, decide what kind of support learners need, and make sure they progress fairly through the education system.

The use of SIAS policy helps in identifying learners who are facing learning barriers and ensures they get personalised support (DBE 2014). This support often comes from a team of professionals, such as therapists (physiotherapists, speech therapists and occupational therapists) and psychologists, who assess learners and help create customised learning plans. These plans are carried out in collaboration with teachers, parents and support staff to promote holistic development (Hayes & Bulat 2017).

Support systems such as School-Based Support Teams (SBSTs) and District-Based Support Teams (DBSTs) help turn policy objectives into practice. School-Based Support Teams work directly within schools to assess needs, coordinate help, and work with families and specialists. District-Based Support Teams support schools more broadly, offering training and helping with policy implementation. Special schools also act as resource hubs, providing specialised lessons, therapies, assistive devices and training for teachers in both special and mainstream schools (DBE 2014).

According to the NPPPR policy, schools must give students with disabilities appropriate assessments and accommodations so they can advance through school and receive formal qualifications (DBE 2017). However, Chataa and Nkengbeza (2019) pointed out challenges in promoting learners without fully preparing them. Disruptive behaviour and low expectations can lead learners to feel hopeless and stop putting in the effort toward their schoolwork (Lawrence-Turner 2011; Lynch 2014). As learners are promoted without fully grasping the content, staff shortages worsen, leaving teachers overwhelmed and affecting the quality of education (Muziransa 2016).

These policies highlight that all learners should be given the right support and resources to access education meaningfully and successfully.

### Assistive devices

Learners with physical disabilities often require the use of assistive devices for support, mobility and prevention of musculoskeletal issues such as poor posture and pain (Edusei & Mji 2019; Schewtschik et al. 2013). Assistive devices play a crucial role in enhancing access and enabling active participation for individuals within their communities, including educational settings (Adugna et al. 2020). However, fewer than one in five individuals in low-income settings have access to the assistive technology they need (Harniss, Samant Raja & Matter 2015; Visagie et al. 2017). Most learners in special schools lack sufficient access to appropriate assistive device because of challenges pertaining to centralised procurement, inadequately trained personnel and a lack of funding for maintenance and replacement (Trafford et al. 2021). It is the view of Solomon et al. (2024) that there is a lack of mandatory enforcement in policy implementation because of the provision that implementation only depends on availability of resources.

### The role of parents

Global and national education policies highlight the vital role of parents in partnering with educators to create learning spaces that nurture students’ academic achievement, social development and emotional well-being (Barger et al. 2019; Guo & Keles 2024; Wong et al. 2018). Yet, most studies on parent involvement tend to focus on educators’ views, with very few giving voice to parents directly. In limited research carried out in Europe, parents generally felt that teachers understood their children’s strengths and offered appropriate support (Paseka & Schwab 2020). Other studies show that parents believe successful inclusive education depends on skilled teachers, meaningful parent participation and a caring attitude (Hanssen & Erina 2022).

Because parents are not usually included in policy discussions and decisions, many inclusive education policies fall short of meeting family needs. Without listening to parents’ experiences and ideas, education systems cannot become truly inclusive or responsive.

This study helps close that gap by focusing on parents’ views of how special schools support children with physical disabilities. Hearing from parents provides valuable insight into how schools deliver education and support services, enabling educators and policymakers to strengthen collaboration and create more inclusive, family-oriented learning environments (Guo & Keles 2024; Kurth et al. 2019).

Understanding these perspectives is essential for turning policy into real, effective support for learners and their families.

## Research methods and design

The study adopted a qualitative approach rooted in the interpretivist perspective. This approach emphasises understanding individuals’ viewpoints, based on the assumption that reality is subjective, multifaceted, and shaped by each person’s unique historical and social context (Kumatongo & Muzata 2021). According to this perspective, reality is shaped by personal experience and varies from person to person.

### Study design

The study undertook an explorative and descriptive, qualitative design using semi-structured interviews (Abu-Halaweh et al. 2021). This approach focuses on representing the views of the participants without considering extensive theoretical interpretation or providing theoretical explanations to the phenomena. This is most suitable, especially in the area where research is limited. For the context of this study, the views of parents regarding support provision at special schools have not been explored sufficiently. Thus, this study design was chosen as it is suitable to strengthen the voices of parents, describe their experiences and share insight towards their views.

### Setting

The study involved parents of learners living with physical disabilities attending special schools in Limpopo Province, South Africa. The rate of individuals with disabilities in the province is estimated at 7 out of every 100 people, with one-quarter of the children experiencing disabilities (Stats SA 2024). Typical of rural provinces, Limpopo Province is among the resource-constrained provinces in South Africa with regard to infrastructure, staff and learning materials. In addition, schools lack adequate support to advance inclusive education from their support structures (Maapola-Thobejane, Ehiane & Prudence 2023). The province only has 35 special schools accommodating learners with various special education needs amid the growing population of children with disabilities. These special schools are widely dispersed geographically among the ten education districts. To simplify data collection, only three nearby special schools were selected because of their proximity to the researcher.

### Recruitment

The researcher communicated with school principals and chairpersons of School Governing Bodies of selected special schools by email, indicating the purpose of the research involving parents of all Grade 7 learners. The researcher was invited to staff meetings to discuss and clarify how parents will be involved and the questions that will be asked in the interview guide. Permission to contact parents was then requested. Letters were prepared (with the researcher’s details included) to inform parents of the research project and to invite them to participate. These letters were issued to parents who visited the schools. To reach more parents, the researcher received authorisation from the school leadership to have contact details retrieved from the South African School Administration and Management System (SASAMS) by the designated personnel. This approach aligned with the *Protection of Personal Information Act (POPIA), Act No. 4* of (RSA 2013) section 18(4). The researcher phoned parents individually. Most of the cellphone numbers were not working; for some, the lines did not exist. In school A, seven parents were reachable out of the 15. In school B, two out of seven, and in school C, two out of 11 were reachable. The recruitment process took place over 4 months from 19 August 2021 to 14 November 2021.

Table 1 shows the demographic characteristics of 11 participants. Of the 11, only one participant was a father and one a grandmother. The majority were single mothers and unemployed (*n* = 6). The median age was 43 years.

**TABLE 1:** Demographic characteristics of parents (*N* = 11).

Participant	Gender	Age of parent (years)	Marital status	Employment status	Relation to learner
P1	Female	30	Single	Unemployed	Mother
P2	Female	53	Single	Unemployed	Mother
P3	Female	42	Single	Unemployed	Mother
P4	Female	55	Single	Unemployed	Mother
P5	Male	52	Married	Educator	Father
P6	Female	35	Married	Unemployed	Mother
P7	Female	33	Single	Unemployed	Mother
P8	Female	45	Married	Admin Clerk	Mother
P9	Female	43	Married	Nurse	Mother
P10	Female	70	Widow	Pensioner	Grandmother
P11	Female	38	Single	Unemployed	Mother

### Study population and sampling strategy

A total of 11 parents who were contacted from the three special schools were interviewed. The purposive non-random sampling method was used to recruit participants through telephone calls. This sampling method enables researchers to select with intention participants who have specific characteristics and experiences that align with the goal of the study (Campbell et al. 2020).

#### Inclusion criteria

Only parents who were reachable via telephone and confirmed to have a child in Grade 7 living with a physical disability from the three selected special schools participated in the study. Parents referred to either a primary or secondary caregiver, regardless of gender, if the individual was registered by the school as the one responsible for the child.

### Ethical considerations

Approval to conduct the study was granted by both the University’s Health Sciences Ethics Committee (Ref:668/2020) and the provincial Department of Education. The research adhered to the ethical principles of conducting studies involving human participants, following the guidelines set out in the Helsinki Declaration version 13 (General Assembly of the World Medical Association 2014). All participants signed informed consent forms, thus agreeing to participate in the study and to have their voices recorded for the interviews.

### Data collection

Data were collected using semi-structured interviews conducted both physically and telephonically. The telephone medium was preferred to reach parents who were widely dispersed and would have involved a high cost for travelling (Ilies et al. 2007). The researcher prepared a spreadsheet to indicate cellphone numbers that were not working, whether consent was given or not, language preference of the parent, the date and time of the appointment to conduct the interview. In cases where parents spoke a language that the researcher was limited to understand, such as Tshivenda, the services of a translator were employed. The researcher informed the translator about the study and coached her on how to probe questions. It is crucial to highlight that the translator was a registered health practitioner and therefore bound by professional ethical standards to maintain confidentiality. While a formal Non-Disclosure Agreement was not signed at the time of the study, the translator was verbally briefed on the importance of protecting the participants’ privacy. Interviews occurred via conference calls only when the translator was available. The researcher sent messages to remind parents about the interview appointment closer to the date and advised them to be in a quiet area. Parents were also informed of the presence of a translator before the interviews. The researcher took notes while the interviews were in progress and audio recorded with the participant’s permission. On average, telephonic interviews lasted 40 min and 50 min for physical (Irvine 2011). Transcripts in Tshivenda were translated to English and transcribed verbatim (Hill et al. 2022). The authors acknowledge the risk of some cultural nuances being lost or altered during transcription; however, this was mitigated by employing a translator proficient in Tshivenda. Transcripts captured not only spoken words but also non-verbal cues such as body language and tone of voice (Denham & Onwuegbuzie 2013). Each transcript was allocated a unique identity number for the sake of confidentiality (Kaiser 2009). Transcripts were sent to parents to verify their statements through WhatsApp and email (Hill et al. 2022).

### Data analysis

Inductive thematic data analysis on ATLAS.ti version 9 was conducted following the six-step approach (Braun & Clarke 2006) to obtain the perceptions of parents concerning their views on how special schools addressed and responded to the unique needs of their children. This approach was most suitable as the study aimed to explore parents’ perceptions, with the aim of making their voices heard. In addition, this approach facilitates themes to emerge directly from the data rather than from pre-existing frameworks. The six steps began with researchers familiarising themselves with the data through repeated readings and note-taking. Initial codes were generated from quotations, which were then grouped into categories and refined into themes following consensus. Finally, the three themes formed the basis of the study’s results and discussion.

Eleven transcripts were uploaded on ATLAS.ti version 9 and analysed retrospectively (Guest, Bunce & Johnson 2006). Two researchers coded the transcripts individually, and the other became a referee during discussions of themes (Hennink, Kaiser & Weber 2019). Data saturation was reached on the coding of the 7th interview, and no new codes emerged from the data. Consequently, thematic analysis was halted. Similar codes were grouped into categories, which then produced overarching themes. A consensus agreement was used as a strategy to determine intercoder reliability (Prieto-González et al. 2021). This strategy allowed back-and-forth and in-depth discussions of themes among the authors (Makwena M Sibuyi; Muziwakhe Tshabalala) yielding credible results.

### Trustworthiness

Trustworthiness was achieved in various ways to ensure rigour relating to credibility, transferability, dependability and confirmability (Johnson, Adkins & Chauvin 2020). Credibility refers to confidence in the truth of the study, and it was established through member checks of 11 participants. Transferability refers to the extent to which the findings can be applied or are relevant to other contexts or groups. This was ensured by providing detailed contextual information about the study setting, interview method and duration. Dependability refers to the consistency and stability of the findings over time and across researchers. This was established through maintaining an audit trail recording study activities for future replication. Confirmability refers to the extent to which the findings of the study are shaped by the participants and not by the researcher. Confirmability was maintained through collaboration with a co-coder (MM; MT), fostering consensus when coding discrepancies emerged (Nowell et al. 2017). The researcher worked with a co-coder to maintain consistency in coding.

## Results

Three overarching themes emerged with inductive thematic data analysis: (1) lack of curriculum differentiation and its effects on the learners’ academic performance, (2) lack of empathy and support in addressing the learners’ challenges, and (3) poor management of assistive devices.

### Theme 1: Lack of curriculum differentiation and its effects on the learners’ academic performance

Parents were of the view that the curriculum was not adapted as it should be to accommodate their children with special educational needs. As a result, because their children were not benefiting from the curriculum, the level of their academic performance was less desirable. This is supported by the following quotations:

‘It seems they teach them the way they do at normal mainstream school from the way I see it. Eish to be honest I am not satisfied with how they teach them. If I’m not mistaken there is no class that my child does not fail. That is why my child is still in Grade 7 at 18 years. Ah last year I started giving up thinking that the child must leave school and stay home with me because I got angry when I saw her reports … It’s like I am wasting time. She gets one report and every time they progress her to be condoned. They should try skills to see if the child can do it. When the child is like this, they want her to write. The child cannot write, even if the child writes eish.’ (P6, Female, 35, Married, Unemployed, Mother)

‘It seems they don’t have skills projects. We don’t know when to be sure because there are those who don’t need to be taught in class but can-do vocational skills. I think if they had skills, my child would have been in that class.’ (P5, Male, 52, Married, Educator, Father)

Parents realised that their children were repeating grades until being progressed to the next grade without meeting the academic promotion requirements and not receiving the necessary support needed. Parents were not satisfied with this practice of their children just being progressed to the next grades because they were worried that there was no learning taking place. This is supported by the following quotations:

‘So, if he has 2 years in the same grade then they progress him. I realised that there is no progress.’ (P5, Male, 52, Married, Educator, Father)

‘The problem is that he repeated Grade 5 a lot of times. But it’s the same with where he is now. He repeated Grade 5 twice; he was just being progressed. He does not understand anything. That is why I want him to do skills.’ (P2, Female, 53, Single, Unemployed, Mother)

‘Previously she failed and had to repeat. She is progressing and I am not happy. Because at the end of the day a progressed child becomes redundant and knows nothing. It means they will keep on progressing her until Grade 12.’ (P8, Female, 45, Married, Admin Clerk, Mother)

### Theme 2: Lack of empathy and support in addressing the learners’ challenges

There was tension between parents and educators as it seemed that educators shifted the responsibility of identifying learning barriers and providing appropriate support onto the parents, while parents expected teachers to possess the skills of recognising barriers and supporting their children accordingly. Educators embraced the learners’ weaknesses more than their strengths and failed to address the learners’ challenges. This is supported by the following quotations:

‘They are complaining that the child is not doing schoolwork. Yes, the teachers are complaining … the child does not want to write. So, they were asking that I should speak to her. This is the third year the teachers have complained. So, I really don’t know if they can’t handle her at school how will I handle her better. I think they should be advising me on what to do but they don’t. Because even if I try to talk to her, she will still be spending a lot of time at school. I see her only during holidays.’ (P6, Female, 35, Married, Unemployed, Mother)

‘So, I’m not even sure what is the main challenge. I also ask how I can assist or support however I can. But some of the teachers complain about her sleeping in class. This one they complain about it … eish [*hopeless*] I think even if she is sleeping in class, they should wake her up. So that she can catch up. So, if they leave her sleeping it’s obvious, she is not learning in class.’ (P8, Female, 45, Married, Admin Clerk, Mother)

### Theme 3: Poor management of assistive devices

Parents complained about the bad condition of their children’s wheelchairs, which posed a risk to their safety. This is supported by the following quotations:

‘That’s another issue with wheelchairs at that school. She started there in 2012, but she has had so many wheelchairs since then. She recently received a wheelchair in January, but you won’t like it when you see it but it’s already broken, and the year has not ended. Imagine since 2012 the child used about four wheelchairs. I don’t know if they are careless or what. When I ask, she does not respond so I asked if other children were using her wheelchairs. She says they play with them. Eish … I am stressed to be honest because to get a wheelchair in hospitals it’s tough. It’s not simple. I cannot stand seeing her in that wheelchair. I rather save money and buy.’ (P6, Female, 35, Married, Unemployed, Mother)

‘We have since applied for a wheelchair at the hospital. We still have not received it. The hand-rests are worn out and they are hurting her. I don’t know what will happen next year at the new school the whole year if we don’t get it.’ (P3, Female, 42, Single, Unemployed, Mother)

The other parent was concerned about her child not putting on callipers while at school because there was no one to monitor compliance in wearing this type of assistive devices. This is supported by the following quotations:

‘You will be doing certain exercises with the child but when she gets to school, she no longer does it because there is no one to monitor. You will just find her walking without callipers because she has no one to assist to put them on.’ (P8, Female, 45, Married, Admin Clerk, Mother)

## Discussion

### Demographic characteristics of participants

The demographic data from this study indicated that many primary caregivers for learners with disabilities were unmarried, unemployed mothers (*n* = 6). The caregiving responsibilities in this context referred to staying home and looking after the needs of the child with disabilities beyond providing an income. These findings are consistent with those of Dhada and Blackbears (2019), who also observed that, in most cases, mothers primarily take on the caregiving responsibilities for children with disabilities (Table 1). The gender disparities in caregiving roles are attributed to societal and cultural perceptions that recognise mothers as more fitting to address the complex emotional and physical needs of children with disabilities (Dempsey & Keen 2008). The caregiving responsibilities that single mothers assume often make securing employment challenging, thereby exacerbating the family’s financial difficulties and contributing to their poverty status (Nicoriciu & Elliot 2023; Rupp & Ressler 2009). Notably, it is rare to have a father assuming the role of a primary caregiver, which is traditionally associated with mothers in many cultural contexts, especially grandmothers. These demographics reflect a changing pattern in parental roles within families managing the responsibilities of caring for a child living with disabilities.

#### Theme 1: Lack of curriculum differentiation and its effects on the learners’ academic performance

Delivery of an inclusive curriculum necessitates differentiation, which, in turn, requires educators to possess knowledge of diverse teaching strategies and the capacity to apply these effectively in response to the learners’ needs. Key indicators of effective differentiation include the use of alternative instructional approaches and strategies to stimulate learner engagement (Tjernberg & Mattson 2014).

According to the views of parents in this study, the curriculum was not sufficiently differentiated to accommodate their children’s individual learning requirements. This finding highlights the challenges associated with implementing a curriculum that is flexible and capable of addressing the diverse needs of learners. Onyishi and Sefotho (2020) attributed this challenge to the lack of curriculum specialists whose responsibility would be to capacitate educators on curriculum-related strategies. This responsibility seems to be expected to be carried over by the SBSTs to mitigate for the knowledge gaps among educators. However, the SBSTs are limited in their functions and responsibilities to support educators and learners because of a lack of training in assuming their roles (Makhalemele & Tlale 2020; Subramoney 2017). In comparison to the South African context, a study conducted in Germany examining parental attitudes and perceptions towards inclusive education and teaching practices found that parents generally reported a strong implementation of innovative teaching methods. These methods were seen as enhancing learner engagement and even benefited learners with learning difficulties (Paseka & Schwab 2020).

The lack of effective curriculum differentiation can be linked to the high number of learners failing and progressing to the next grade without support systems being in place. The views of parents in this study concurs with those of parents of children with intellectual disabilities in Lesotho who participated in semi-structured interviews regarding the provision of inclusive education. In the above-mentioned study, parents reported that their children acquired neither academic nor social competencies and resulting in learners dropping out of school (Tseeke 2024).

Parents in this study preferred their children to do vocational skills because they realised they were struggling with the mainstream education. However, special schools did not cater for the needs of these affected learners. A study by Basister and Valenzuela (2021) in the Philippines revealed that learners with special educational needs receiving vocational education often acquired skills that enabled them to contribute economically and achieve financial independence. However, in the South African context, the implementation of vocational education faces significant challenges, as reported through semi-structured interviews with teachers involved in vocational education programmes for learners with special needs in Cape Town. These challenges included but were not limited to inadequate training for educators, insufficient infrastructure, and limited access to resources necessary for practical skill development (Solomon et al. 2024). This finding suggests that without vocational education, learners risk losing the opportunity to achieve financial independence. Consequently, this increases their reliance on parental support and government resources, as they are unprepared for employment to sustain themselves.

Although there is limited research in the South African context on parents’ views regarding curriculum differentiation, evidence from international studies highlights the need to invest in educator training and the development of school-based support systems to enable inclusive and differentiated teaching practices. South Africa’s schools have inclusive education policies in place, but they lack the necessary resources to effectively implement vocational training and curriculum differentiation. This gap perpetuates the disparity between the goals of the policies and the actual experiences in the classroom.

#### Theme 2: Lack of empathy and support in addressing learners’ challenges

The theme showed a lack of empathy on the side of educators when engaging the learners and parents. Parents perceived educators to be complaining about the challenges their children experience in the classroom instead of collaborating with parents to work out solutions. Parents became hopeless and frustrated, unsure of where to go for support. Tjernberg and Mattson (2014) advocate for personalised instruction to identify areas where the learner needs to be supported. Parents in Russia who participated in a study that explored their views on inclusive education for their children with special educational needs viewed humanity as one of the crucial aspects necessary for implementing inclusive education (Hanssen & Erina 2022). Humanity involves understanding the world from the child’s perspective to acknowledge their struggles, strengths and delivery of support in a caring, non-judgemental way (UNESCO 2020). Parents in Germany (in the study mentioned in Theme 1) attested to the need for instruction that is personalised and accompanied by empathy in inclusive classrooms. These parents reported that educators built strong connections with learners and prioritised the learners’ interests (Paseka & Schwab 2020). Unlike in this study, parents are disappointed that children are not engaged optimally in the classroom. The educators could be portraying this negative attitude towards parents and learners because they do not feel supported by their institutions in performing their duties. As a result, teachers may unintentionally express frustration or disengagement, which parents interpret as a lack of empathy and commitment. This study concurs with Watermeyer, McKenzie, and Kelly (2024) that emotional and structural challenges hinder teachers from effectively implementing inclusive education. Thus, the negative perceptions held by parents are frequently symptoms of deeper systemic issues, where educators’ emotional well-being and access to professional support are not given sufficient priority. To enhance learner engagement and build stronger relationships with parents, schools must invest in comprehensive support systems that equip teachers with the emotional resilience and professional confidence needed to adopt inclusive practices.

#### Theme 3: Poor management of assistive devices

Parents reported that their children’s assistive devices were often worn out and difficult to replace. One of the main challenges was the long wait list at public institutions, particularly for wheelchairs. Furthermore, as many of the children stay in hostels during the school term, families often have to wait until the end of the term to replace items such as crutches at their nearest referral hospital.

Many schools struggle to maintain and replace assistive devices because of constraints in budgets and the centralisation of procurement processes. This approach leads to allocations of assistive devices being made once-off and excluding additional costs associated with the maintenance and replacement of wheelchairs and crutches for example (World Bank 2023). The views of parents in this study concur with a study involving parents of learners with physical disabilities in Iceland investigating the use, impact of mobility devices on activities and participation and satisfaction with the service delivery process of mobility devices (Gudjonsdottir & Gudmundsdottir 2023). Parents in the study reported dissatisfaction with the lack of follow-up and maintenance of the devices their children use at special schools. The lack of proper follow-up for assistive devices in schools can result in delays in necessary adjustments or replacements. The school’s environmental conditions significantly contribute to the rapid wear and tear of these devices. For example, uneven playground surfaces, learners using the same devices for play and sports, and friends improperly propelling learners in wheelchairs along lengthy corridors connecting classrooms and hostels all exacerbate device deterioration (Gudjonsdottir & Gudmundsdottir 2023). These factors highlight the need for regular maintenance checks, user training and tailored environmental modifications to ensure the durability and safe use of assistive devices for learners.

### Limitations

The findings of this study are limited to parents whom the researcher was able to reach by telephone. The views expressed by parents are inherently subjective, shaped by their deep concern for their children’s well-being and prospects. As a result, it is important to observe that these views should not be generalised to all parents. In addition, the study lacked gender diversity as only one male father participated. Thus, the voices and views of fathers were underrepresented.

Because of limited time and resources, the study did not include parents of learners from other special schools in the province. Although the interview guide was not piloted, it was shared with participants in advance, allowing them to engage with the questions, seek clarification, and prepare their responses. The study employed the services of a translator, which could have caused some information to be misinterpreted.

## Conclusion

This study provides important insights into parents’ views on how special schools addressed the unique needs of learners with physical disabilities. Parents identified significant gaps in the use of varied teaching methods. A key issue was the limited ability of educators to adapt the curriculum effectively to meet the diverse needs of learners. This limitation highlights an urgent need for professional development to equip teachers with the skills to personalise learning to improve both academic and social outcomes for learners with special educational needs.

The findings also revealed that educators often focused predominantly on learners’ challenges and limitations rather than recognising and responding to their individual strengths and interests. This approach can hinder learner motivation and participation. Cultivating empathy among educators is crucial in encouraging them to understand and appreciate each learner’s unique experiences and capabilities, which fosters a more inclusive and engaging learning environment. Furthermore, the study uncovered systemic barriers that restricted the full implementation of inclusive education in special schools. There was a notable lack of effective management of assistive devices, which are essential for supporting learners with physical disabilities. In addition, the shortage of appropriate materials and inadequate infrastructure severely limited opportunities for vocational skills training, an important pathway to independence and future employment.

Despite the inclusive education policy’s goals, learners were frequently excluded from meaningful learning experiences because of these institutional and policy-level challenges. Addressing these issues would require comprehensive strategies that enhance educators’ capacity, improve resource availability, and strengthen infrastructure and assistive technology management. Only through such coordinated efforts can special schools truly provide equitable, inclusive, and empowering education for all learners with special educational needs.

### Recommendations

Based on the findings, the following recommendations can be helpful.

Curriculum specialists within the special schools’ district should provide educators with professional development on how to modify the curriculum to meet the diverse needs of learners with special education needs. This includes adapting lessons to accommodate different learning styles and providing more individualised support to learners.

The school principals alongside the School Based Support Teams (SBTs) should strengthen programmes where parents would be encouraged to be involved in all decision-making processes pertaining to their children. These programmes will ensure that their concerns are heard and that their input is incorporated into school policies to promote inclusive education.

The provincial Department of Education should ensure that special schools, particularly as resource centres, are adequately funded and equipped to provide the necessary support for learners with special educational needs. This includes providing resources for vocational education, accessible learning materials and staff training to support curriculum differentiation.

The SBT, including therapists such as physiotherapists, speech therapists and occupational therapists, should collaborate in implementing the SIAS process for the early identification of learners’ interests and strengths. Through comprehensive assessments, SBT can guide curriculum stream selection to better align learning experiences with each learner’s abilities. These assessments should provide evidence-based recommendations to ensure that learners who experience difficulties with the mainstream academic curriculum are not unnecessarily retained in their current grades. Instead, they should be directed towards skill-based subjects that promote greater engagement and support their overall development.
